# Efficacy of Different Irrigant Activation Systems on Debris and Smear Layer Removal: A Scanning Electron Microscopy Evaluation

**DOI:** 10.1155/2023/9933524

**Published:** 2023-09-20

**Authors:** Ronald Wigler, Moran Herteanu, Yuval Wilchfort, Anda Kfir

**Affiliations:** ^1^Department of Endodontology, The Goldschleger School of Dental Medicine, Tel Aviv University, Tel Aviv, Israel; ^2^The Goldschleger School of Dental Medicine, Tel Aviv University, Tel Aviv, Israel

## Abstract

**Background:**

Irrigation is an essential component of root canal treatment to enable cleaning beyond the reach of mechanical instruments. The study aimed to assess and compare the efficacy of different final irrigation protocols, including sonic- and ultrasonic-powered irrigant-activation systems, on debris and smear layer removal in the coronal, middle, and apical thirds of straight oval root canals.

**Materials and Methods:**

Straight oval root canals of 60 human mandibular incisors were prepared to size 40.04 and divided into four groups (*n* = 15) according to the final irrigation protocols: (a) Eddy sonic activation (b) endosonic passive ultrasonic irrigation (PUI), (c) irrisafe PUI, and (d) manual syringe and needle irrigation with no additional activation, which served as control. After the treatment procedures, the roots were split and observed using scanning electron microscopy. The presence of remaining debris and smear layer at the coronal, mid-root, and apical thirds of the canals were evaluated using a score system and statistically analyzed using multinominal models with significance level set at *p* < 0.05.

**Results:**

None of the final irrigation protocols completely removed all debris and smear layer from all root canals. When the syringe and needle were used without activation, more debris and smear layer were found in the apical third of the canals. Activation of the final irrigant with each of the three devices significantly reduced the presence of debris in the apical third, compared to the syringe and needle final irrigation, with no difference among the three activation devices. Eddy and irrisafe activation also significantly reduced the residual smear layer in the apical third, compared to syringe and needle alone, while the reduction in the remaining smear layer by endosonic activation did not reach the significance level.

**Conclusions:**

Removal of debris and smear layer from the apical part of the root canal by syringe and needle irrigation alone may be significantly improved by using sonic or ultrasonic activation of the final irrigant. Endosonic activation was less effective in removal of smear layer from the apical part of the canals compared to the other two activation systems.

## 1. Introduction

Shaping and cleaning of the root canal system are the most important components of successful root canal treatment [1, 2]. With the current endodontic instruments at our disposal and the complexity of the canal system, it is impossible to thoroughly shape and clean it [3]. According to studies, approximately 10% to 50% of the surface of the root canal system is untouched by instruments [4–7]. Therefore, irrigation is an essential component of root canal treatment to enable cleaning beyond the reach of root canal instruments in areas that were not mechanically touched [2, 3]. However, manual irrigation with a syringe and needle is not able to reach beyond 1.5 mm from the needle tip and is of limited efficiency when complex canal anatomies are considered [8, 9]. Since effective cleaning procedure requires proper removal of all debris and smear layer, different machine-assisted irrigant activation techniques have been introduced to overcome the limitations of manual syringe and needle irrigation. Following mechanical instrumentation [2, 3, 9–11].

A recently published systematic review showed that sonic and ultrasonic activation techniques improved intracanal cleanliness compared to conventional syringe and needle irrigation, but none rendered the canal walls free of smear layer and debris [9].

EndoSonic PS (SelectD, Seoul, Korea) is a new ultrasonic-powered irrigant activation system made of autoclavable flexible polymer with tip sizes 20/0.03 and 25/0.03, which was recently introduced. According to the manufacturer, it is designed to work in straight and curved root canals and allows for the safe removal of debris and smear layer from the canal system (SelectD). In cases of breakage, the manufacturer claims that the broken part can be easily flushed out by irrigation (SelectD). However, there is no published data on the efficacy of the EndoSonic PS (SelectD) ultrasonic activation system. Therefore, this study aimed to assess and compare the efficacy of EndoSonic PS (SelectD) with two popular irrigant activation systems, the Eddy sonic activation system (VDW, Munich, Germany), and the Irrisafe passive ultrasonic irrigation (PUI) system (Satelec Acteon, Merignac-Cedex, France), and compare their efficiency in the removal of debris and smear layer to that of syringe and needle irrigation with no additional activation.

The null hypothesis was that there would be no difference in the removal of smear layer and debris between the three irrigant activation systems and manual syringe and needle irrigation.

## 2. Materials and Methods

The study design was based on that of Urban et al. [2] and was conducted in accordance with the Declaration of Helsinki and approved by the Institutional Ethics Committee (IEC No. 55.19).

### 2.1. Tooth Selection

A total of 60 human mandibular incisors, 18–20 mm in length, with intact apices and no previous treatments, which were extracted for periodontal reasons, were selected. Each tooth was mounted on a Protrain endodontic system (Simit Dental, Mantova, Italy) and radiographed from buccal and proximal directions to evaluate the angle of the curvature of the canals [12] and to verify a single oval root canal [13]. Only straight root canals (angle of curvature < 5°) and those with a long:short canal diameter ratio of ≥2.5 at 5 mm from the apex [13] were selected for the study.

An access cavity was prepared using diamond burs, and patency was established using a #10 K-file (Mani, Utsunomiya, Japan). The length of each canal was determined under a dental microscope (Karl Kaps, Asslar, Germany) by inserting the file until the tip was just visible at the apical foramen. The incisal edges of the crowns were horizontally cut and ground using diamond burs to obtain a total length of 17 and 16 mm working length (WL).

The apices of the roots were covered with wax to provide a closed system (Baseplate wax, St. George Technology, Wilmington, NC, USA).

### 2.2. Instrumentation and Irrigation Protocol

All root canals were prepared by an endodontic specialist to #40/.04 using 2-shaped instruments (Micro-Mega, Besançon, France).

Throughout the mechanical preparation, the canals were irrigated with 1 mL of 4% NaOCl solution following each instrument. After mechanical preparation was completed, a rinse with 5 mL of 17% EDTA was performed, and the roots were then randomly assigned to three experimental groups and one control group (*n* = 15).

In each group, a total volume of 6 mL of 4% NaOCl was used for final irrigation with one of the final irrigant activation protocols that were tested in the study. Irrigating solutions were delivered in all groups using a 30-G needle (NaviTip; Ultradent, South Jordan, UT, USA), which was inserted 1 mm from WL without binding.

### 2.3. Final Irrigant Activation Protocols

#### 2.3.1. Eddy Sonic Activation (N = 15)

Sonic activation was performed with a 28 mm, polymer tip (Eddy) (#0.25, taper 0.06) (VDW, Munich, Germany) powered by an airscaler handpiece (W&H, Bürmoos, Austria) at 1 mm from the WL. Setting: 6,000 Hz. The canals were irrigated with a total volume of 6 mL NaOCl and three activation cycles (2 mL of NaOCl per cycle) of 20 s each were used.

#### 2.3.2. Endosonic PS Passive Ultrasonic Activation (N = 15)

PUI was performed with a 23.5 mm, polymer tip (#0.25, taper 0.03) (EndoSonic PS; SelectD, Seoul, Korea) powered by a Satalec P5 Newtron ultrasonic system (Satelec Acteon) at 1 mm from the WL. Setting: 30% of the scale, resulting in approximately 30,000 Hz. The canals were irrigated with a total volume of 6 mL of NaOCl, and three activation cycles (2 mL of NaOCl per cycle) of 20 s each were performed.

#### 2.3.3. Irrisafe Passive Ultrasonic Activation (N = 15)

PUI was performed with a 25 mm, stainless steel, non-cutting wire (#0.25, taper 0.00) (Irrisafe; Satelec Acteon, Merignac-Cedex, France) powered by a Satalec P5 Newtron ultrasonic system (Satelec Acteon) at 1 mm from the WL. Setting: 30% of the scale, resulting in approximately 30,000 Hz. The canals were irrigated with a total volume of 6 mL of NaOCl, and three activation cycles (2 mL of NaOCl per cycle) of 20 s each were used.

#### 2.3.4. Syringe and Needle with No Activation Control

Final irrigation was performed with 6 mL of 4% NaOCl. No activation was applied.

Finally, all canals were rinsed with 5 mL of distilled water and dried with paper points (FKG Dentaire, La Chaux de Fonds, Switzerland).

### 2.4. Scanning Electron Microscope Analysis

The roots were split longitudinally using a diamond disc (Horico, Berlin, Germany) and a chisel. Prior to splitting, a gutta perch cone size 40/.04 (FKG Dentaire) was placed in the canals to avoid contamination with dentine particles [14]. Only one part of the root was randomly selected and coded for evaluation of the presence of remaining debris and smear layer on the canal walls. In order to avoid a later potential bias in the selection of fields for photomicrography, horizontal marks were made on the split surface of the root walls at the middle part of the coronal, middle, and apical thirds of the canals using a sharp scalpel. These marks, which are easy to identify during electron microscopy at low magnification, were later used to objectively locate, with no bias, the center of each root canal section and used for field selection for photomicrography under scanning electron microscopy (SEM) (see below) [15]. The teeth were then fixed with glutaraldehyde, dehydrated through a graded series of ethanol solutions, subjected to critical point drying, and gold-plated for SEM examination (JSM-25S; Jeol, Tokyo, Japan).

The field to be photographed was determined as the root canal surface at the midpoint between the above-mentioned scalpel markings. This was done to avoid any bias in field selection. Photomicrographs at ×200 and ×1,000 magnification were taken at that midpoint to assess the presence of debris (magnification ×200) and smear layer (magnification ×1,000). The images were randomly coded and analyzed two times at 4-week intervals by two experienced blinded observers who underwent a calibration process using a set of 20 representative images.

**Figure 1:**
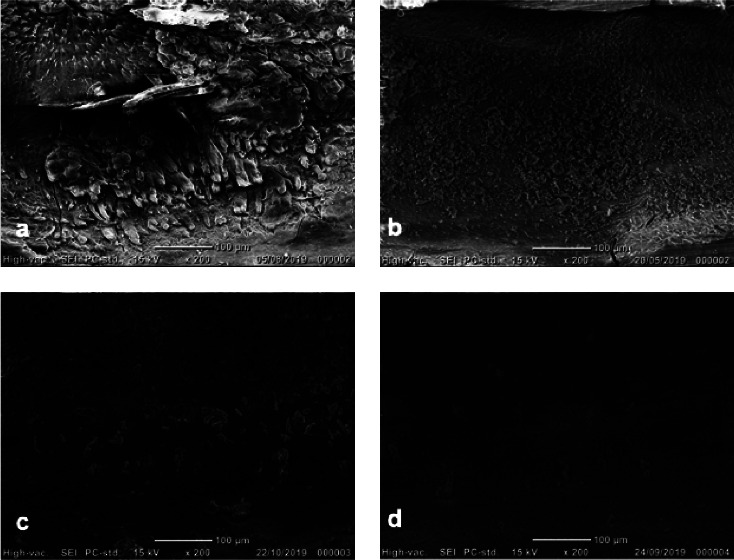
Illustrative images of remaining debris covering the root canal wall (magnification ×200). (a) Score 5. (b) Score 4. (c) Score 3. (d) Score 2.

**Figure 2:**
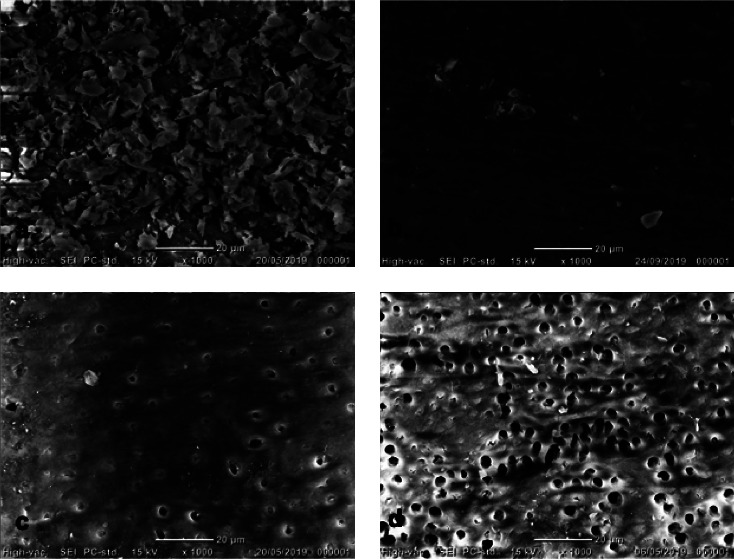
Illustrative images of remaining smear layer covering the root canal wall (magnification × 1,000). (a) Score 5. (b) Score 4. (c) Score 3. (d) Score 2.

Debris was defined as pulp tissue remnants, dentine chips, and particles loosely attached to the root canal walls [16]. The smear layer was defined as a surface film of debris consisting of dentin particles, pulp tissue remnants, and bacterial components retained on dentine after root canal instrumentation [16]. Each image was rated on a scale of 1–5 for the presence of debris/smear layer using the rating system used in previous studies [2, 15, 16]. The criteria for debris scoring were the following: Score 1: canal walls are clean with only a few debris particles. Score 2: a few small accumulations covering less than 25% of the canal wall. Score 3: accumulations covering 25%–50% of the canal wall. Score 4: accumulations covering 50%–75% of the canal wall. Score 5: accumulations covering >75% of the canal wall with debris. The criteria for smear layer scoring were the following: Score 1: open dentine tubules; no smear layer. Score 2: some open dentine tubules; a small amount of smear layer. Score 3: few open dentine tubules; homogenous smear layer along almost the entire root canal wall. Score 4: no open dentine tubules; the canal wall is covered with a homogenous smear layer. Score 5: A thick homogenous smear layer covers the canal wall. All scoring procedures were performed individually by each examiner. When disagreement in scoring occurred, it was discussed, and an agreement was reached. Figures 1 and 2 are presented to illustrate the score system.

SAS 9.4 Program (SAS Institute, Cary, NC, USA) was used to analyze the data. Intra- and inter-examiner reliability were verified by the Kappa test. Multinomial mixed models with repeated measures for each tooth were used to assess differences between thirds of the root canal. Multinomial models were used to assess differences between thirds of the root canal and methods of irrigation. The significance level was set at *p* ≤ 0.05.

## 3. Results

The kappa values for inter- and intra-observer agreement were 0.888 and 0.910, respectively. The scores of remaining debris and smear layer when different final irrigation/activation methods were applied are presented in Table 1, and the differences between the methods in cleaning the apical third of the canal are presented in Tables 2 and 3. Since there were no significant differences between all four groups in the coronal and mid-root segments of the canals, this comparison is only presented in the text with no table presentation.

Images of different amounts of remaining debris and smear layer covering the root canal wall are presented in Figures 1 and 2, respectively, to illustrate the score systems for the evaluation of debris and smear layer.

None of the irrigation techniques completely removed all debris and smear layer from all root canals.

No significant difference was found in the coronal and mid-root segments between any of the activation protocols and syringe and needle activation, thus only the analysis of the results of the apical third is presented in detail (Tables 2 and 3).

When each of the three irrigant activation devices was used, there was no difference between them in the coronal, mid-root, and apical segments of the root canal, in terms of debris removal. When syringe and needle irrigation were used with no activation, the apical third of the canals contained significantly more debris than the coronal and mid-root segments (*p*=0.028). The efficacy of debris removal from the apical part of the canal by each of the activation protocols was significantly better than that of irrigation with a syringe and needle without activation: *p* < 0.001, *p*=0.002, and *p* < 0.001, for Eddy, endosonic, and irrisafe, respectively. The three activation protocols did not differ from each other in respect to the removal of debris from the apical third of the canal (Table 2).

When removal of the smear layer from the canal walls was concerned, the smear layer scores in the apical third were significantly higher than in the coronal and mid-root sections when Endosonic or syringe and needle were used (*p* < 0.001 and *p*=0.001, respectively). No difference in smear layer scores between the canal thirds was found when Eddy and Irrisafe activation protocols were used.

When comparing the efficacy of smear layer removal in the apical third of the canals, Eddy and Irrisafe protocols were more effective than syringe and needle irrigation without activation: *p*=0.005 and *p*=0.026 for Eddy and irrisafe protocols, respectively. The difference in smear layer removal in the apical part of the canals between the endosonic protocol and syringe and needle irrigation did not reach the significance level (*p*=0.090). No difference was found between the Eddy and Irrisafe irrigant activation protocols in their ability to remove the smear layer (Table 3).

## 4. Discussion

Scanning electron microscopy has been often used to evaluate the efficacy of cleaning root canals [2, 16, 17]. However, this method has a major potential disadvantage, as the selection of fields to be evaluated may be subject to bias. In this study, this issue was addressed by a strict predetermination of the field to be photographed, the midpoint between two clearly detectable marks, thus avoiding any potential bias in field selection.

Many new endodontic instruments have been introduced in the last decade; nevertheless, studies have shown that at least 10% of the main root canal walls remain untouched by the instrument and more than 50% in the case of anatomical variations of the root canal that contain recesses and isthmuses [4–7, 18, 19].

Recent studies on instruments that claimed to mechanically clean the root canal three-dimensionally showed that even in straight root canals, more than 10% of the dentine walls remained untouched [20, 21]. Therefore, effective irrigation is essential to clean untouched walls and areas that are not accessible to mechanical instrumentation [2, 3, 19]. Additional activation of the irrigants may increase the removal of debris and smear layer from the canal system, compared to manual irrigation with a syringe and needle [2, 9, 22]. There is currently a debate in the literature as to which activation technique is more effective for cleaning the canals, but there is widespread agreement that all activation techniques are more effective in comparison to syringe and needle irrigation alone [9].

The most common irrigation method among endodontists seems to be PUI. Approximately 50% of them use this method in addition to manual irrigation with a syringe and needle [23, 24]. PUI with a metallic tip has some disadvantages, namely the contact of the metal tip with the canal walls that constrains the tip movement, dampens the energy, and might remove small amounts of dentine in an uncontrolled manner during irrigant activation and trigger the formation of a new undesirable smear layer [14, 24–26]. Sonic activation instruments such as the Eddy activator have smooth, none-cutting, flexible tips and may have an advantage compared to metal tips used for PUI since they don't stop when in contact with the canal walls and are not able to remove dentine [14, 24, 26].

PUI activates the irrigant by generating acoustic microstreaming, and the instrument's oscillation gives rise to shear stress forces next to the canal walls [3, 27, 28]. Sonic activation is not able to produce acoustic streaming and has a quarter of PUI's oscillation frequency [28, 29], but its cleaning ability is assumed to be the same as PUI because it has a higher amplitude and its tip makes three-dimensional orbital movements while PUI oscillates transversely in one plane [28, 30, 31]. In this study, the Eddy sonic activation and Irrisafe ultrasonic activation presented similar efficiency in removing both debris and smear layer from the coronal and mid-root areas and even from the more challenging cul-de-sac area of the apical part of the root canal.

In this study, EndoSonic PS, a new polymeric tip for PUI, was also tested as a potential instrument for the removal of debris and smear layer. It performed well in the coronal and mid-root areas of the canal and was as effective as the other two methods in removal of debris from the apical part, yet its performance in smear layer removal from the apical part of the canal was less effective than the other two activation protocols.

Even though the activation protocols resulted in relatively clean canals, similar to Urban et al. [2], complete removal of all debris from the canal walls of all samples was not obtained. In the coronal and mid-root parts of the canal, no difference was found between syringe and needle final irrigation and irrigation assisted by any of the activation methods; thus, the null hypothesis was accepted in regard to these sections of the root canals.

However, concerning debris removal in the apical part of the canal, the null hypothesis had to be rejected, as all three of the activation techniques were significantly better than manual irrigation with a syringe and needle alone.

Regarding smear layer removal, Urban et al. [2] showed that Eddy sonic activation and PUI performed equally, and both performed significantly better than manual irrigation with a syringe and needle alone. The present results are in agreement with these findings when the apical part of the canal is concerned, yet in the coronal and mid-root areas, no difference was found between the final irrigant activation methods and the syringe and needle alone. Thus, in the coronal and mid-root parts of the canal, the null hypothesis was also accepted as far as smear layer removal is concerned. On the other hand, in the apical area, significant differences were found between syringe and needle irrigation alone and irrigant activation by the Eddy sonic device or the Irrisafe ultrasonic device; thus, the null hypothesis had to be rejected as far as the apical part is concerned.

The difference between the present results and those of Urban et al. [2] could be explained by the fact that EDTA was used in the present study at the final stage of instrumentation, before final irrigation was performed with NaOCl with or without activation. Urban et al. [2] did not use a chelating solution in their study. It has previously been established that irrigation with NaOCl alone cannot dissolve inorganic dentin particles, and therefore it cannot effectively remove the smear layer that forms during instrumentation [32, 33]. This difference between the present results and those of Urban et al. [2] emphasizes the need to use a chelating solution to accomplish effective smear layer removal.

Overall, relatively clean canal walls were found in the present study. This can be attributed to the 40/.04 preparation size combined with a thin needle that was used for irrigation, which allowed sufficient streaming and exchange of the irrigation solution [2, 34].

A limitation of the present study was that it was performed only in straight root canals. Only a few studies examined debris and smear layer removal from curved root canals, presenting contradictory results [17, 22, 35, 36]. Further research is needed to examine the effectiveness of ultrasonic activation of polymeric tips *vs*. metal tips in cleaning curved root canals.

## 5. Conclusions

Removal of debris and smear layer from the apical part of the root canal by syringe and needle irrigation alone may be significantly improved by using sonic or ultrasonic activation of the final irrigant. Endosonic activation was less effective in removal of smear layer from the apical part of the canals compared to the other two activation systems.

## Figures and Tables

**Table 1:** Remaining debris and smear layer scores with different irrigation/activation methods.

Debris scores															
	Apical third					Mid-root third					Coronal third				
	1	2	3	4	5	1	2	3	4	5	1	2	3	4	5
Eddy	6	9	0	0	0	7	7	1	0	0	6	6	2	1	0
EndoSonic PS	5	8	2	0	0	6	8	1	0	0	8	5	2	0	0
Irrisafe	6	8	1	0	0	12	3	0	0	0	9	5	1	0	0
Needle	0	7	4	2	2	4	6	4	1	0	3	11	1	0	0
Smear layer scores															
	1	2	3	4	5	1	2	3	4	5	1	2	3	4	5
Eddy	5	3	4	3	0	7	3	2	2	1	8	3	3	1	0
EndoSonic PS	3	2	5	5	0	7	5	2	1	0	9	3	3	0	0
Irrisafe	5	2	4	2	2	5	5	3	1	1	11	0	3	1	0
Needle	1	1	2	10	1	2	6	3	4	0	10	2	1	2	0

Number of samples with a given score. Needle: Syringe and needle irrigation with no additional activation. SL Scores, smear layer scores.

**Table 2:** Differences in remaining debris between irrigation/activation methods in the apical third of the canal (*p* values).

	Eddy	Endosonic	Irrisafe	Needle
Eddy	—	NS	NS	<0.001
Endosonic	NS	—	NS	0.002
Irrisafe	NS	NS	—	<0.001
Needle	<0.001	0.002	<0.001	—

Needle: Syringe and needle irrigation with no additional activation. NS: *p* value > 0.05.

**Table 3:** Differences in remaining smear layer between irrigation/activation methods in the apical third of the canal (*p* values).

	Eddy	Endosonic	Irrisafe	Needle
Eddy	—	NS	NS	0.005
Endosonic	NS	—	NS	NS
Irrisafe	NS	NS	—	0.026
Needle	0.005	NS	0.026	—

Needle: Syringe and needle irrigation with no additional activation. NS: *p* value > 0.05.

## Data Availability

The data are available in the text (Tables 1–3).
